# Global, regional, and national burdens of attention deficit hyperactivity disorder in adolescents and young adults aged 10–24 years from 1990 to 2021: A trend analysis

**DOI:** 10.1371/journal.pone.0341076

**Published:** 2026-02-23

**Authors:** Bo Yuan, Bo Ao, Sajid Ali, Qiong Cai, Yanan Chen, Youliang Huang

**Affiliations:** 1 School of Management, Beijing University of Chinese Medicine, Beijing, China; 2 Department of Pediatrics, China-Japan Friendship Hospital, Beijing, China; 3 University of Education, Multan Campus, Multan, Pakistan; 4 Department of Social Medicine and Health Education, School of Public Health, Peking University, Beijing, China; 5 National Institute of Chinese Medicine Development and Strategy, Beijing University of Chinese Medicine, Beijing, China; Johns Hopkins School of Medicine and Kennedy Krieger Institute, UNITED STATES OF AMERICA

## Abstract

**Background:**

Attention Deficit Hyperactivity Disorder (ADHD) is a major neurodevelopmental disorder among adolescents and young adults (AYAs) worldwide. However, there is still insufficient understanding of the burden and trends of this condition. This study aims to assess the trends in the global, regional, and national burden of ADHD among AYAs aged 10–24 years from 1990 to 2021.

**Methods:**

Based on the Global Burden of Diseases, Injuries, and Risk Factors Study (GBD) 2021, our study reports estimates of the incidence, prevalence, and disability-adjusted life-years (DALYs) of ADHD among AYAs at the global, regional, and national levels, including the corresponding rates and 95% uncertainty intervals (UIs). We also analyzes the trends in the burden of ADHD from 1990 to 2021 from both global and local perspectives, specifically by Estimated Annual Percentage Change (EAPC) and Average Annual Percentage Changes (AAPCs). Additionally, these global trends are further examined according to age, sex, and Socio-Demographic Index (SDI).

**Results:**

Globally, the incidence of ADHD among AYAs decreased slightly from 12.61 per 100,000 population in 1990 to 11.89 per 100,000 population in 2021, with an EAPC of −0.61% (95% CI −0.79 to −0.43) and AAPCs of −0.17% (95% CI −0.28 to −0.05). The prevalence rate decreased slightly from 2,381.82 per 100,000 population in 1990–2,173.48 per 100,000 population in 2021, with an EAPC of −0.58% (95% CI −0.63 to −0.53) and AAPCs of −0.44% (95% CI −0.47 to −0.42). The rate of DALYs decreased from a 1990 rate of 30.31 per 100,000 population to 26.56 per 100,000 population in 2021, with an EAPC of −0.58% (95% CI −0.63 to −0.52) and AAPCs of −0.44% (95% CI −0.47 to −0.42). In terms of gender, incidence, prevalence and rates of DALYs were higher in males than in females during the same period. In terms of age, the incidence rate originated only from the 10–14 years age group, and only prevalence and DALYs rates were present in the 15–19 years age group and 20–24 years age group and the trend analysis results were correlated with the age group. According to SDI quintiles, incidence, prevalence, and rates of DALYs for ADHD had the highest increases from 1990 to 2021 in areas with a High-middle SDI or High SDI. However, the relationship between incidence, prevalence, and DALYs rates and SDI was nonlinear, and regionally, Australasia had the highest incidence, prevalence, and DALYs rates in 2021, with Western Europe and East Asia having the largest increases in incidence, prevalence, and DALYs rates. In terms of countries, Australia has the highest incidence, prevalence and DALYs rates in 2021, while the UK, Spain and China have the highest rate increases.

**Conclusions:**

Over the past 30 years, there has been a general downward trend in the incidence and prevalence of ADHD and in the rate of DALYs worldwide. However, phased studies have shown less homogeneous trends in recent years, which may be related to changes in the level of socioeconomic development, diagnostic criteria, and therapeutic approaches. Therefore, it is necessary to continue to promote research on the accuracy and universality of ADHD diagnosis and treatment in the future, with a view to further reducing the global health burden of ADHD.

## 1 Introduction

Attention deficit hyperactivity disorder (ADHD), also known as hyperactivity disorder or attention deficit syndrome, is a neurodevelopmental disorder primarily characterized by symptoms of inattention and hyperactivity, which occur during childhood and are disproportionate to the individual’s age [1]. These core symptoms are often associated with other features, such as emotional instability, cognitive impairments, and learning difficulties. In 2010, ADHD was included in the Global Burden of Diseases, Injuries, and Risk Factors Study (GBD) [2], and it is estimated that ADHD contributes to a direct economic burden of billions of dollars annually worldwide [3]. However, a recent reassessment of the GBD 2019 study indicates that the prevalence and burden of ADHD may have been underestimated [4].

Despite the growing recognition of ADHD, there is still a limited number of studies focusing on the disease burden of ADHD based on the GBD database. One notable study specifically addresses the ADHD burden in the Middle East and North Africa [5], along with several other studies that include ADHD as part of mental health disorders. Most existing research has focused on the GBD 2019 data without conducting trend analyses. This study aims to investigate the trends in the incidence, prevalence, and disability-adjusted life-years (DALYs) associated with ADHD globally from 1990 to 2021. The trends are stratified by age, gender, and Socio-Demographic Index (SDI), and regional and national level trends are reported to provide valuable insights for healthcare policy and resource allocation.

## 2 Materials and methods

### 2.1 Study population and data collection

GBD 2021, published by the Institute for Health Metrics and Evaluation (IHME) based in the United States, represents the most comprehensive and detailed global study on diseases, injuries, and risk factors. The data from this study are publicly available as open-source information. The GBD 2021 encompass 204 countries and territories, providing estimates of the burden of 371 diseases and injuries, including ADHD, from 1990 to 2021. The methodology and estimation processes of GBD 2021, as well as its foundational approach, have been outlined in prior systematic analysis studies of the GBD [6,7]. Importantly, all data utilized in this research are drawn from publicly accessible databases, and as such, no additional ethical approval was required.

While the United Nations defines adolescence as the period between 10 and 19 years [8], the definition of young adulthood remains less clear. A study suggests that defining the young adulthood phase as 10–24 years is more consistent with common understandings of this life stage [9]. In the GBD 2021 database, age groups for adolescents are categorized into 10–14 years, 15–19 years, and 20–24 years, along with a combined group for those aged 10–24 years. Given this, and to align with both the database structure and general demographic definitions, this study defines adolescents and young adults (AYAs) as individuals aged 10–24 years.

The study focuses on analyzing trends in three primary indicators: incidence, prevalence, and DALYs rate. DALYs serves as a comprehensive measure of disease burden by combining years of life lost due to premature mortality and years lived with disability. Using data from GBD 2021, the DALYs for ADHD in the AYAs group are calculated and trends are examined. Furthermore, GBD 2021 also calculates the SDI for each country, which reflects the social and economic development of regions based on factors like education level, per capita income, and fertility rates. The SDI is categorized into five levels: low, low-middle, middle, high-middle, and high [6,7].

In this research, data on ADHD incidence, prevalence, and DALYs were extracted from the GBD 2021 database for three age groups (10–14 years, 15–19 years, and 20–24 years), as well as for 21 geographically close and epidemiologically similar regions. These data are further analyzed by 204 countries and territories and classified according to SDI. The resulting estimates include the corresponding 95% uncertainty intervals (UIs).

### 2.2 Statistical analysis

The data analysis methods used in this study are based on the approaches of Zhang Jing et al [10]and Leiwen Fu et al [11]. To provide a more comprehensive understanding of the disease burden of ADHD, a descriptive analysis was conducted, grouping ADHD by gender and age categories at the global, regional, national, and SDI levels. Additionally, the study visually presents the incidence, prevalence, and DALYs rates of ADHD in the 10–24-year-old adolescent group in 2021 at the national level, as well as the impact of different SDI levels across regions and countries.

This study employs both the Estimated Annual Percentage Change (EAPC) and the Joinpoint regression analysis to examine the overall and segment-specific trends in ADHD. EAPC is a method used to evaluate the average change in disease burden over a specified time interval [12]. In this study, EAPC was applied to analyze the overall trends in ADHD incidence, prevalence, and DALYs rates among AYAs. It was assumed that the natural logarithms of ADHD incidence, prevalence, and DALYs rates in AYAs follow a linear regression model, as shown in Equation (1), where γ refers to the natural logarithm of the incidence, prevalence, and DALYs rates, and x represents the year, where *α* is the intercept, *β* is the slope, and *ε* is the error term. After transformation, Equations (2) and (3) are derived [13]. If the estimated EAPC and the upper bound of its 95% confidence intervals (CIs) are both less than 0, the trend is considered to be decreasing. If the estimated EAPC and the lower bound of its 95% CIs are both greater than 0, the trend is considered to be increasing. If neither condition is met, the trend is considered stable.


γ=α+βx+ε (1)

ln(Rate)=α+βx+ε (2)

EAPC=100×(exp(β)−1) (3)

The Annual Percentage Changes (APCs) is calculated as the geometric weighted average of various annual percentage changes in the regression analysis. Age-specific rates and their Average Annual Percentage Changes (AAPCs) are calculated using a linear regression model with the natural logarithm of the ratio on a logarithmic scale as the dependent variable and the year as the independent variable. AAPCs represent a summary measure of the trend over a predefined time period and are computed as the weighted average of APCs, allowing us to describe the average APCs over the study period with a single number. The value of AAPCs reflects the percentage change per year, which can represent an increase, decrease, or no change.

The Joinpoint regression analysis model is a widely used statistical method in epidemiological studies to assess temporal trends in disease incidence or mortality [14]. In this study, the Joinpoint regression model was used to evaluate the local temporal trends in the disease burden of ADHD. This method fits several line segments, known as “joinpoints”, on a logarithmic scale, testing the model using the Monte Carlo permutation method. The final model was selected using the weighted Bayesian Information Criterion (BIC) and the suggestions provided by the Joinpoint regression software. Using this model, the APCs, AAPCs, and their 95% CIs for incidence, prevalence, and DALYs trends were calculated. If the estimated value of APCs/AAPCs and the lower bound of its 95% CIs are both greater than 0, the trend is considered to be increasing. Conversely, if the estimated value of APCs/AAPCs and the upper bound of its 95% CIs are both less than 0, the trend is considered to be decreasing. If neither condition is met, the trend is considered stable. All statistical analyses were performed using RStudio software (version 4.4.1) and Joinpoint regression software (version 4.9.1.0).

## 3 Results

### 3.1 Incidence

In 2021, an estimated 224,405 new cases of ADHD were reported among AYAs globally. The incidence of ADHD slightly decreased from 12.61 per 100,000 population in 1990 to 11.89 per 100,000 population in 2021. The overall trend showed an EAPC of −0.61% (95% CI: −0.79 to −0.43), indicating an average annual decline in age-standardised incidence. Local trend analysis, using AAPCs, revealed a decline of −0.17% (95% CI: −0.28 to −0.05) undergoing the fifth joinpoint. The incidence showed an upward trend from 1990 to 1998 and from 2013 to 2021, with varying decreases in 2001–2006, 2006–2009, and 2009–2013, while it remained stable between 1998 and 2001.

Regarding gender, both male and female groups showed a slight decline in ADHD incidence, with males having a significantly higher incidence rate than females. From 1990 to 2021, the age-standardised incidence for males declined by 0.63% (95% CI: −0.81 to −0.45) annually, while females showed a similar decline of 0.61% (95% CI: −0.79 to −0.42). Locally, males exhibited a roughly declining trend, with an AAPCs of −0.19% (95% CI: −0.30 to −0.07), while females showed an increase in incidence from 1990 to 1999 and from 2013 to 2021, with declines from 1999 to 2002, 2002–2006, and 2006–2010, and stability between 2010 and 2013.

**Fig 1:**
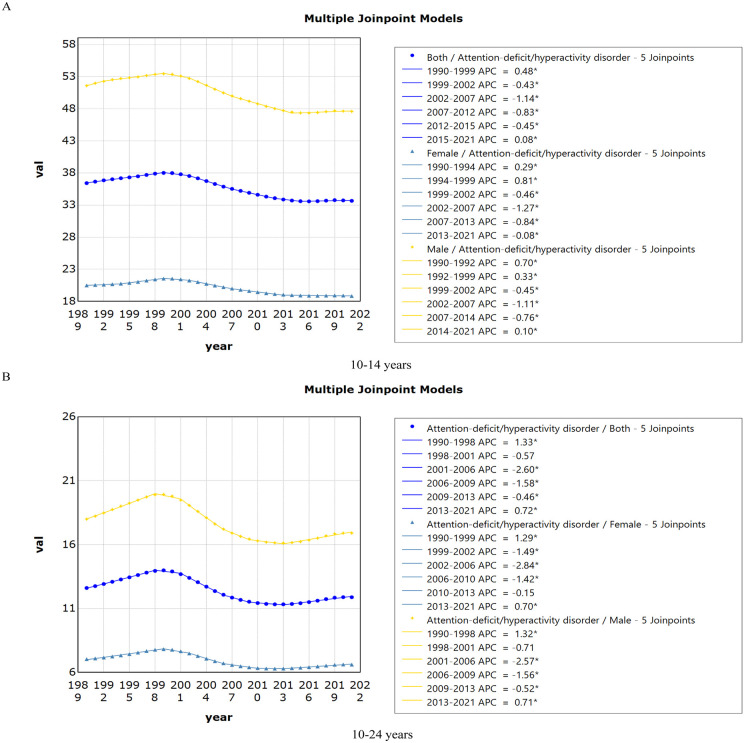
The joinpoint regression analysis on the rate of Incidence of ADHD among 10-14 years (A) and among 10-24 years (B) AYAs. Abbreviations:* Indicates that the APCs is significantly different from zero at the alpha = 0.05 level. ADHD = attention deficit hyperactivity disorder. AYAs = adolescents and young adults.

For age groups, ADHD incidence in AYAs mainly stems from the 10−14 age group, hence the analysis focused on this group. The EAPC for ADHD incidence in 10–14-year-olds was −0.44% (95% CI: −0.52 to −0.35), reflecting an average annual decline in age-standardised incidence. Locally, the AAPCs post-fifth joinpoint was −0.25% (95% CI: −0.30 to −0.20), with statistically significant downward trends. The incidence increased from 1990 to 1999 and from 2015 to 2021, but declined during several subsequent periods: 1999–2002, 2002–2007, 2007–2012, and 2012–2015(Fig 1).

ADHD incidence in AYAs varied across SDI levels, with higher incidence in high and high-middle SDI regions. In general, higher SDI correlates with higher incidence. Overall, SDI regions with middle SDI showed the largest average annual decrease in age-standardised incidence (EAPC = −0.44%, 95% CI: −0.51 to −0.38), while high SDI regions showed the greatest increase (EAPC = 0.21%, 95% CI: 0.17 to 0.25). The highest AAPCs was observed in high-middle SDI regions (0.11%, 95% CI: 0.10 to 0.12), while low-middle SDI regions had the lowest AAPCs (−0.04%, 95% CI: −0.04 to −0.04).

Regionally, Western Europe experienced the highest increase in ADHD incidence, with an average annual increase of 0.50% (95% CI: 0.46 to 0.54), while North Africa and Middle East saw the largest decline, with an average annual decrease of 0.69% (95% CI: −0.82 to −0.55). Local trend analysis showed East Asia with the highest AAPCs (0.33%, 95% CI: 0.32 to 0.35), Tropical Latin America showed the lowest AAPCs (−0.10%; 95% CI −0.11 to −0.08) (Table 1).

**Table 1:** The case number and rate of incidence of ADHD among AYAs in 1990 and 2021 by SDI quintiles and by GBD regions,with EAPC and AAPCs from 1990 to 2021.

	1990		2021		EAPC (95% CIs)	AAPCs (95% CIs)
	Number (95% UIs)	Rate (95% UIs)	Number (95% UIs)	Rate (95% UIs)		
**Global**	195088.50 (133791.77 to 282314.95)	12.61 (8.65 to 18.25)	224405.15 (152449.42 to 323235.73)	11.89 (8.08 to 17.12)	−0.61 (−0.79 to −0.43)	−0.17 (−0.28 to −0.05)
**Sex**						
Male	141502.22 (97542.66 to 205065.64)	18.00 (12.41 to 26.09)	163566.87 (111410.35 to 235645.85)	16.91 (11.52 to 24.36)	−0.63 (−0.81 to −0.45)	−0.19 (−0.30 to −0.07)
Female	53586.29 (36404.88 to 77879.95)	7.04 (4.78 to 10.23)	60838.28 (40853.17 to 88533.18)	6.61 (4.44 to 9.62)	−0.61 (−0.79 to −0.42)	−0.16 (−0.33 to 0.01)
**Age group**						
10-14 years	195088.50 (133791.77 to 282314.95)	36.42 (24.98 to 52.70)	224405.15 (152449.42 to 323235.73)	33.66 (22.87 to 48.49)	−0.44 (−0.52 to −0.35)	−0.25 (−0.30 to −0.20)
15-19 years	0.00 (0.00 to 0.00)	0.00 (0.00 to 0.00)	0.00 (0.00 to 0.00)	0.00 (0.00 to 0.00)	NA	NA
20-24 years	0.00 (0.00 to 0.00)	0.00 (0.00 to 0.00)	0.00 (0.00 to 0.00)	0.00 (0.00 to 0.00)	NA	NA
**GBD regions**						
East Asia	61461.14 (42696.44 to 90392.58)	16.51 (11.47 to 24.29)	63009.81 (43347.54 to 90433.33)	25.93 (17.84 to 37.21)	0.30 (−0.23 to 0.83)	0.33 (0.32 to 0.35)
Southeast Asia	16404.44 (11507.41 to 23854.10)	11.06 (7.76 to 16.08)	16381.86 (11004.53 to 24148.09)	9.58 (6.44 to 14.12)	−0.51 (−0.54 to −0.48)	−0.05 (−0.05 to −0.05)
Oceania	274.92 (187.89 to 409.86)	13.14 (8.98 to 19.59)	515.34 (352.18 to 768.15)	12.78 (8.73 to 19.05)	−0.21 (−0.27 to −0.16)	−0.01 (−0.02 to −0.01)
Eastern Europe	5044.34 (3346.24 to 7481.27)	10.68 (7.09 to 15.84)	3825.25 (2536.15 to 5680.40)	11.59 (7.69 to 17.22)	−0.32 (−0.89 to 0.25)	0.04 (0.03 to 0.05)
Central Europe	3195.77 (2156.33 to 4706.29)	10.95 (7.39 to 16.12)	1920.11 (1295.22 to 2832.79)	10.59 (7.14 to 15.62)	−0.25 (−0.50 to 0.01)	−0.01 (−0.01 to −0.00)
High-income Asia Pacific	5610.26 (3735.98 to 8286.53)	13.32 (8.87 to 19.67)	3451.77 (2346.80 to 5126.92)	13.22 (8.99 to 19.64)	0.06 (−0.06 to 0.19)	−0.00 (−0.01 to 0.00)
Central Asia	2181.14 (1467.26 to 3235.88)	11.00 (7.40 to 16.32)	2509.61 (1688.61 to 3725.57)	11.34 (7.63 to 16.84)	−0.56 (−0.84 to −0.27)	0.01 (0.01 to 0.02)
Western Europe	12009.01 (8257.73 to 17430.21)	14.61 (10.05 to 21.20)	12186.16 (8181.97 to 17877.01)	16.91 (11.35 to 24.80)	0.50 (0.46 to 0.54)	0.08 (0.08 to 0.09)
Caribbean	3058.45 (2130.63 to 4437.52)	28.64 (19.95 to 41.55)	3266.63 (2238.40 to 4827.09)	28.84 (19.76 to 42.61)	−0.17 (−0.29 to −0.06)	0.02 (0.01 to 0.02)
Southern Latin America	2050.47 (1383.55 to 3005.64)	15.49 (10.45 to 22.71)	2168.28 (1442.03 to 3131.74)	14.14 (9.40 to 20.42)	−0.43 (−0.48 to −0.37)	−0.05 (−0.05 to −0.05)
Andean Latin America	2887.16 (1970.01 to 4206.14)	23.45 (16.00 to 34.17)	3684.26 (2513.98 to 5375.54)	21.34 (14.56 to 31.14)	−0.40 (−0.44 to −0.36)	−0.07 (−0.07 to −0.07)
Australasia	1443.56 (1008.34 to 2037.60)	30.00 (20.96 to 42.35)	1935.93 (1373.80 to 2650.75)	33.74 (23.95 to 46.20)	0.14 (0.02 to 0.25)	0.13 (0.12 to 0.13)
High-income North America	12331.38 (8239.55 to 18153.11)	20.16 (13.47 to 29.67)	15520.65 (10295.61 to 22957.99)	21.77 (14.44 to 32.21)	−0.07 (−0.19 to 0.06)	0.06 (0.05 to 0.07)
Central Latin America	8641.61 (5838.63 to 12518.01)	15.93 (10.76 to 23.07)	9395.80 (6462.18 to 13690.10)	14.45 (9.94 to 21.05)	−0.34 (−0.40 to −0.27)	−0.05 (−0.05 to −0.04)
North Africa and Middle East	15899.92 (10990.92 to 23369.94)	14.60 (10.09 to 21.46)	20768.04 (14229.91 to 30542.63)	12.80 (8.77 to 18.82)	−0.69 (−0.82 to −0.55)	−0.06 (−0.06 to −0.06)
Central Sub-Saharan Africa	1076.88 (711.94 to 1587.5)	6.22 (4.11 to 9.17)	2825.29 (1867.88 to 4164.85)	6.29 (4.16 to 9.27)	0.00 (−0.03 to 0.03)	0.00 (0.00 to 0.00)
South Asia	21920.66 (14513.58 to 32989.49)	6.55 (4.34 to 9.86)	30574.74 (20362.88 to 45215.92)	5.81 (3.87 to 8.60)	−0.37 (−0.42 to −0.33)	−0.02 (−0.02 to −0.02)
Tropical Latin America	10771.94 (7379.40 to 15598.93)	22.51 (15.42 to 32.59)	9903.81 (6719.53 to 14621.91)	19.58 (13.29 to 28.91)	−0.34 (−0.47 to −0.21)	−0.10 (−0.11 to −0.09)
Eastern Sub-Saharan Africa	3996.55 (2624.99 to 5928.60)	6.44 (4.23 to 9.56)	8783.59 (5775.16 to 13030.69)	6.04 (3.97 to 8.96)	−0.12 (−0.16 to −0.08)	−0.01 (−0.02 to −0.01)
Southern Sub-Saharan Africa	993.56 (646.79 to 1477.77)	5.82 (3.79 to 8.65)	1248.45 (813.50 to 1858.27)	5.72 (3.73 to 8.52)	−0.24 (−0.38 to −0.10)	−0.00 (−0.00 to −0.00)
Western Sub-Saharan Africa	3835.35 (2519.20 to 5779.95)	6.41 (4.21 to 9.66)	10529.79 (6878.97 to 15685.99)	6.53 (4.26 to 9.72)	0.12 (0.09 to 0.15)	0.00 (0.00 to 0.00)
**SDI quintiles**						
Middle SDI	77632.31 (53703.41 to 112656.21)	14.15 (9.79 to 20.53)	81636.94 (55779.12 to 117491.59)	14.77 (10.09 to 21.26)	−0.41 (−0.67 to −0.15)	0.03 (0.03 to 0.03)
High-middle SDI	42207.70 (29154.05 to 61347.48)	14.87 (10.27 to 21.62)	40884.62 (28003.35 to 58532.24)	18.10 (12.40 to 25.91)	−0.16 (−0.52 to 0.19)	0.11 (0.10 to 0.12)
Low-middle SDI	33006.92 (22102.80 to 47875.55)	9.13 (6.11 to 13.24)	43865.43 (29789.28 to 63859.24)	7.94 (5.39 to 11.55)	−0.49 (−0.52 to −0.46)	−0.04 (−0.04 to −0.04)
High SDI	30802.45 (20705.9 to 44895.49)	15.73 (10.57 to 22.92)	32631.12 (21902.56 to 47597.99)	17.58 (11.80 to 25.65)	0.08 (−0.02 to 0.18)	0.06 (0.06 to 0.07)
Low SDI	11221.26 (7470.82 to 16602.15)	7.21 (4.80 to 10.67)	25164.33 (16852.82 to 37293.18)	6.81 (4.56 to 10.10)	−0.09 (−0.12 to −0.06)	−0.01 (−0.01 to −0.01)

Abbreviations: AYAs, adolescents and young adults. SDI, socio-demographic index. GBD, Global Burden of Diseases, Injuries, and Risk Factors Study. EAPC, estimated annual percentage change. UIs, uncertainty intervals. CIs, confidence intervals. AAPCs, average annual percentage changes.

**Fig 2:**
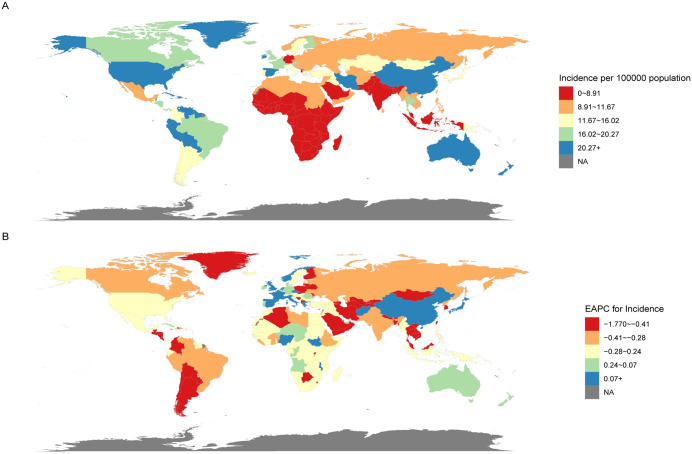
Global map of 2021 incidence (A) and the trend (EAPC) in incidence (B) of ADHD among AYAs. Abbreviations: EAPC = estimated annual percentage change. ADHD = attention deficit hyperactivity disorder. AYAs = adolescents and young adults.

At the national level, Fig 2 summarizes the global incidence and EAPC of ADHD in AYAs in 2021. Overall, Spain showed the highest increase in ADHD incidence, with an age-standardised incidence increasing by 1.15% (95% CI: 0.93 to 1.37) annually, while Qatar showed the largest decrease with a reduction of 1.77% (95% CI: −2.40 to −1.14). Considering the weighted APCs, China exhibited the highest AAPCs (0.35%, 95% CI: 0.33 to 0.37), whereas Grenada displayed the lowest AAPCs (−0.22%, 95% CI: −0.23 to −0.21).

**Fig 3:**
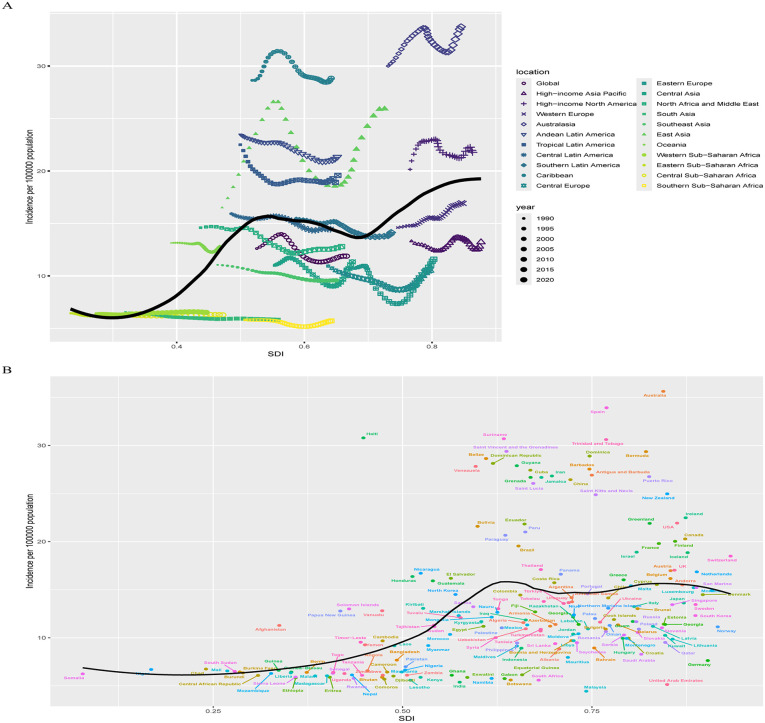
The association between the incidence of ADHD among AYAs and SDI at the regional level from 1990 to 2021 (A) and national level at 2021 (B). Abbreviations: ADHD = attention deficit hyperactivity disorder. AYAs = adolescents and young adults. SDI = socio-demographic index.

Regarding the relationship between incidence and SDI regions, a nonlinear pattern emerged. As SDI increases, ADHD incidence fluctuates between rising and falling (Fig 3A). Between 1990 and 2021, notable regional differences in incidence changes were observed. East Asia, High-Income North America, Tropical Latin America, Andean Latin America, Australasia, the Caribbean, and Oceania had higher-than-expected incidence rates, with the East Asia region demonstrated the most substantial fluctuations, with its incidence rate consistently exceeding projected levels throughout the study period. Conversely, Western Europe, High-Income Asia Pacific, Eastern Europe, Central Europe, Central Asia, Southeast Asia, and Southern Sub-Saharan Africa exhibited lower-than-expected incidence rates.

At the national level, the relationship between ADHD incidence and SDI regions also displayed a nonlinear trend (Fig 3B). High disease burden was observed not only in developed countries but also in underdeveloped countries. However, high SDI countries showed a greater number of countries exceeding expected incidence rates compared to low SDI countries, with notable differences in expected rates. Countries like Australia and Spain had the highest incidence above expectations, while countries like Malaysia exhibited the largest deviations below expected incidence rates.

### 3.2 Prevalence

In 2021, an estimated 41,030,400 cases of ADHD were reported among AYAs globally. The prevalence of ADHD slightly decreased from 2,381.82 per 100,000 population in 1990–2,173.48 per 100,000 population in 2021. The overall trend showed an EAPC of −0.58% (95% CI: −0.63 to −0.53), reflecting an average annual decline in age-standardised prevalence. Local trend analysis revealed an AAPCs of −0.44% (95% CI: −0.47 to −0.42) undergoing the third joinpoint, showing a statistically significant downward trend. The prevalence increased from 1990 to 1993, followed by varying degrees of decline from 1993 to 2000, 2000–2015, and 2015–2021.

By gender, both male and female AYAs showed slight decreases in prevalence, with males exhibiting a significantly higher prevalence rate than females. From 1990 to 2021, the age-standardised prevalence for males decreased by 0.60% (95% CI: −0.65 to −0.55) annually, while for females, it decreased by 0.57% (95% CI: −0.64 to −0.50). Locally, males exhibited a roughly declining trend with an AAPCs of −0.45% (95% CI: −0.49 to −0.42), while females exhibited an increasing trend from 1990 to 2000, followed by declining trends from 2000 to 2008, 2008–2013, 2013–2017, and 2017–2021.

**Fig 4:**
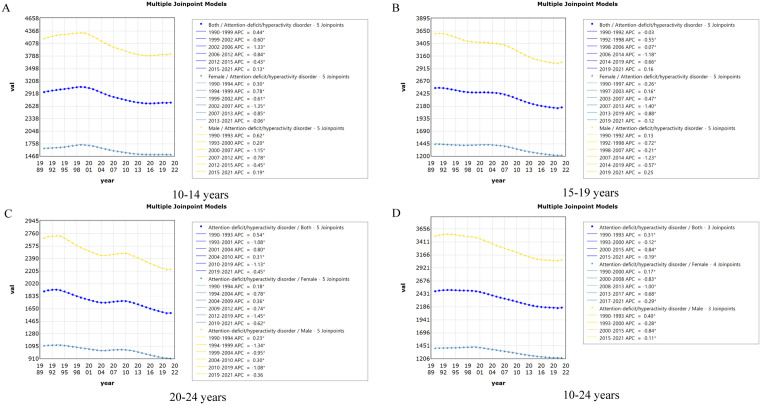
The joinpoint regression analysis on the rate of Prevalence of ADHD among 10-14 years (A), 15-19 years (B), 20-24 years (C) and 10-24 years (D) AYAs. Abbreviations:* Indicates that the APCs is significantly different from zero at the alpha = 0.05 level. ADHD = attention deficit hyperactivity disorder. AYAs = adolescents and young adults.

Regarding age groups, the prevalence of ADHD followed similar trends across all groups. The 20−24 age group showed the most pronounced decline in prevalence, with an EAPC of −0.61% (95% CI: −0.67 to −0.55), followed by the 15−19 age group (EAPC = −0.59%, 95% CI: −0.65 to −0.53) and the 10−14 age group (EAPC = −0.49%, 95% CI: −0.57 to −0.40). Local trend analysis showed that the AAPCs for the 20−24 age group was the smallest (−0.60%, 95% CI: −0.65 to −0.55), while the 10−14 age group had the smallest decline (−0.28%, 95% CI: −0.33 to −0.24) (Fig 4).

Prevalence varied across SDI regions, in general, a higher SDI is positively associated with an elevated disease prevalence. The overall trend showed the largest decline in age-standardised prevalence in middle SDI regions (EAPC = −0.45%, 95% CI: −0.51 to −0.38)annually, while high SDI regions showed the largest increase (EAPC = 0.21%, 95% CI: 0.18 to 0.25). Local trend analysis, considering weighted APCs, revealed that middle SDI regions had the lowest AAPCs (−7.32%, 95% CI: −7.92 to −6.73), while high SDI regions had the highest AAPCs (9.36%, 95% CI: 9.12 to 9.60).

Regionally, Western Europe showed the highest increase in age-standardised prevalence, with an annual increase of 0.23% (EAPC = 0.23%, 95% CI: 0.21 to 0.25), while North Africa and Middle East exhibited the largest decrease (EAPC = −0.41%, 95% CI: −0.50 to −0.32). Local trend analysis indicated that East Asia had the highest AAPCs (17.39%, 95% CI: 15.33 to 19.45), while North Africa and Middle East had the lowest AAPCs (−8.64%, 95% CI: −9.04 to −8.25) (Table 2).

**Table 2:** The case number and rate of prevalence of ADHD among AYAs in 1990 and 2021 by SDI quintiles and by GBD regions,with EAPC and AAPCs from 1990 to 2021.

	1990		2021		EAPC (95% CIs)	AAPCs (95% CIs)
	Number (95% UIs)	Rate (95% UIs)	Number (95% UIs)	Rate (95% UIs)		
**Global**	38398506.35 (26610745.85 to 53946735.15)	2481.82 (1719.94 to 3486.75)	41030399.52 (28462267.14 to 57975717.34)	2173.48 (1507.72 to 3071.12)	−0.58 (−0.63 to −0.53)	−0.44 (−0.47 to −0.42)
**Sex**						
Male	27667421.52 (19269512.59 to 38723994.28)	3519.63 (2451.31 to 4926.15)	29726996.66 (20751061.57 to 41933018.69)	3072.73 (2144.93 to 4334.40)	−0.60 (−0.65 to −0.55)	−0.45 (−0.49 to −0.42)
Female	10731084.82 (7449317.66 to 15271196.55)	1409.94 (978.75 to 2006.45)	11303402.86 (7838549.66 to 16024973.15)	1228.19 (851.71 to 1741.22)	−0.57 (−0.64 to −0.50)	−0.45 (−0.47 to −0.42)
**Age group**						
10-14 years	15834220.19 (10909054.45 to 22869096.07)	2955.90 (2036.48 to 4269.15)	18088249.21 (12387514.85 to 26056649.29)	2713.36 (1858.21 to 3908.68)	−0.49 (−0.57 to −0.40)	−0.28 (−0.33 to −0.24)
15-19 years	13178128.85 (8996273.09 to 18444408.06)	2537.07 (1731.97 to 3550.94)	13461409.41 (9223790.42 to 18800544.50)	2157.35 (1478.22 to 3013.00)	−0.59 (−0.65 to −0.53)	−0.53 (−0.58 to −0.48)
20-24 years	9386157.31 (6495393.11 to 13117416.69)	1907.41 (1319.97 to 2665.67)	9480740.90 (6551628.25 to 13210785.40)	1587.64 (1097.13 to 2212.28)	−0.61 (−0.67 to −0.55)	−0.60 (−0.65 to −0.55)
**GBD regions**						
East Asia	14040913.45 (9905451.50 to 19382812.74)	3772.62 (2661.47 to 5207.92)	10719734.86 (7627915.16 to 14858873.54)	4411.22 (3138.92 to 6114.49)	0.01 (−0.14 to 0.16)	17.39 (15.33 to 19.45)
Oceania	46605.30 (32585.38 to 66662.70)	2227.88 (1557.68 to 3186.69)	89555.13 (62753.66 to 127793.83)	2220.90 (1556.24 to 3169.19)	−0.02 (−0.03 to −0.02)	−0.29 (−0.34 to −0.23)
Eastern Europe	994796.85 (686057.73 to 1438260.58)	2106.34 (1452.63 to 3045.32)	704769.70 (483607.68 to 1020624.68)	2136.00 (1465.71 to 3093.29)	−0.06 (−0.13 to 0.02)	1.15 (0.99 to 1.30)
Southeast Asia	2924521.33 (2038812.94 to 4010723.93)	1971.15 (1374.18 to 2703.26)	3121748.58 (2160951.74 to 4347018.15)	1825.49 (1263.65 to 2541.98)	−0.26 (−0.27 to −0.25)	−4.75 (−4.80 to −4.69)
Central Asia	400367.14 (273704.00 to 563554.94)	2018.87 (1380.17 to 2841.75)	449853.64 (307418.71 to 633322.91)	2033.02 (1389.31 to 2862.17)	−0.08 (−0.12 to −0.04)	0.45 (0.35 to 0.54)
Caribbean	637213.27 (447679.68 to 855303.54)	5967.10 (4192.24 to 8009.38)	679871.32 (473604.14 to 922360.34)	6001.95 (4181.01 to 8142.66)	0.01 (0.00 to 0.02)	1.17 (1.01 to 1.34)
High-income North America	2313438.72 (1610525.24 to 3235434.50)	3781.78 (2632.73 to 5288.97)	2982820.80 (2028186.62 to 4269081.82)	4184.70 (2845.41 to 5989.23)	0.15 (0.07 to 0.23)	13.89 (12.81 to 14.96)
Andean Latin America	530826.51 (365300.29 to 753887.66)	4312.03 (2967.42 to 6124.00)	740584.94 (512434.18 to 1046915.93)	4289.70 (2968.18 to 6064.06)	−0.03 (−0.03 to −0.02)	−0.77 (−0.82 to −0.72)
High-income Asia Pacific	1131200.18 (772406.81 to 1629502.96)	2685.14 (1833.47 to 3867.97)	686886.30 (475125.46 to 971548.53)	2631.25 (1820.06 to 3721.70)	−0.03 (−0.06 to 0.01)	−1.43 (−1.79 to −1.07)
Central Europe	600430.79 (413927.65 to 854456.77)	2056.39 (1417.64 to 2926.39)	371414.22 (256407.36 to 529988.71)	2047.91 (1413.79 to 2922.27)	−0.04 (−0.08 to −0.01)	−0.21 (−0.28 to −0.14)
Australasia	296083.26 (213584.89 to 404313.71)	6154.14 (4439.40 to 8403.73)	365245.54 (269711.30 to 471654.98)	6366.30 (4701.12 to 8221.04)	0.10 (0.07 to 0.13)	7.13 (6.65 to 7.62)
Southern Latin America	345590.56 (235153.34 to 476954.60)	2610.84 (1776.52 to 3603.26)	391176.73 (271584.17 to 544407.26)	2550.63 (1770.84 to 3549.76)	−0.11 (−0.12 to −0.10)	−2.08 (−2.17 to −1.98)
Central Sub-Saharan Africa	179380.78 (122118.46 to 258981.97)	1036.41 (705.57 to 1496.33)	467313.46 (318012.16 to 674556.48)	1039.77 (707.58 to 1500.88)	0.00 (−0.01 to 0.01)	0.10 (0.09 to 0.12)
Tropical Latin America	2053607.30 (1431249.62 to 2933159.26)	4290.90 (2990.52 to 6128.68)	2137312.91 (1494297.05 to 3029755.36)	4225.89 (2954.52 to 5990.43)	0.15 (0.06 to 0.23)	−2.76 (−4.18 to −1.33)
Central Latin America	1570572.34 (1085951.44 to 2255429.80)	2894.77 (2001.55 to 4157.05)	1853918.42 (1300332.29 to 2649300.75)	2850.73 (1999.49 to 4073.77)	−0.01 (−0.03 to 0.01)	−1.45 (−1.89 to −1.01)
Western Europe	2163041.68 (1548653.90 to 2988050.77)	2631.42 (1884.00 to 3635.08)	2003016.85 (1387459.61 to 2808295.87)	2779.06 (1925.01 to 3896.33)	0.23 (0.21 to 0.25)	4.81 (4.63 to 4.99)
Western Sub-Saharan Africa	641184.61 (432896.56 to 949950.77)	1071.43 (723.38 to 1587.39)	1770795.77 (1184193.55 to 2592027.61)	1097.38 (733.86 to 1606.31)	0.10 (0.09 to 0.12)	0.85 (0.84 to 0.86)
South Asia	3870075.96 (2624199.42 to 5460958.28)	1157.08 (784.59 to 1632.72)	5933390.74 (4036530.63 to 8471525.92)	1128.30 (767.59 to 1610.95)	0.03 (−0.03 to 0.08)	−0.83 (−1.03 to −0.62)
North Africa and Middle East	2828537.24 (1979312.40 to 4021680.95)	2597.23 (1817.45 to 3692.80)	3814148.43 (2688950.28 to 5275522.44)	2350.10 (1656.81 to 3250.53)	−0.41 (−0.50 to −0.32)	−8.64 (−9.04 to −8.25)
Eastern Sub-Saharan Africa	652395.84 (444396.83 to 945844.98)	1051.68 (716.38 to 1524.73)	1518993.07 (1036606.22 to 2201177.93)	1044.44 (712.76 to 1513.50)	−0.01 (−0.02 to 0.00)	−0.22 (−0.24 to −0.20)
Southern Sub-Saharan Africa	177723.22 (120844.22 to 254938.93)	1040.31 (707.37 to 1492.30)	227848.12 (155135.66 to 326814.71)	1044.48 (711.16 to 1498.15)	−0.02 (−0.04 to 0.00)	0.15 (0.14 to 0.17)
**SDI quintiles**						
Middle SDI	15965100.07 (11146369.13 to 22356178.39)	2908.95 (2030.95 to 4073.45)	14893911.40 (10438960.62 to 21021559.54)	2694.54 (1888.57 to 3803.12)	−0.45 (−0.51 to −0.38)	−7.32 (−7.92 to −6.73)
High SDI	5873603.34 (4073080.95 to 8237333.80)	2998.92 (2079.62 to 4205.78)	6104052.38 (4231026.73 to 8625745.68)	3289.27 (2279.96 to 4648.12)	0.21 (0.18 to 0.25)	9.36 (9.12 to 9.60)
Low SDI	1864887.58 (1268001.24 to 2681440.82)	1198.05 (814.59 to 1722.62)	4363381.98 (2995371.78 to 6304865.75)	1181.16 (810.84 to 1706.71)	0.01 (−0.01 to 0.02)	−0.44 (−0.53 to −0.35)
Low-middle SDI	5781048.95 (3947113.37 to 8270841.52)	1598.24 (1091.23 to 2286.58)	8265936.50 (5709351.06 to 11813631.74)	1495.43 (1032.91 to 2137.27)	−0.16 (−0.18 to −0.14)	−3.36 (−3.57 to −3.16)
High-middle SDI	8871601.84 (6200831.69 to 12342415.49)	3126.18 (2185.05 to 4349.22)	7359596.16 (5210895.63 to 10345477.50)	3258.19 (2306.93 to 4580.07)	−0.18 (−0.27 to −0.09)	2.33 (1.22 to 3.44)

Abbreviations: AYAs, adolescents and young adults. SDI, socio-demographic index. GBD, Global Burden of Diseases, Injuries, and Risk Factors Study. EAPC, estimated annual percentage change. UIs, uncertainty intervals. CIs, confidence intervals. AAPCs, average annual percentage changes.

**Fig 5:**
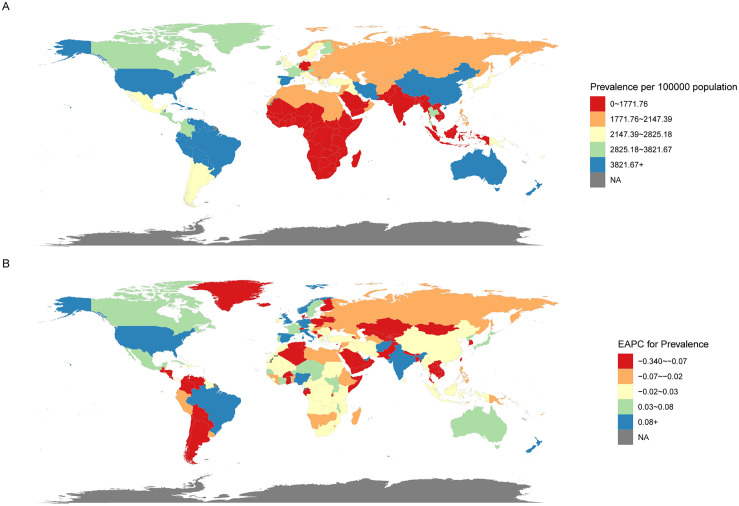
Global map of 2021 prevalence (A) and the trend (EAPC) in prevalence (B) of ADHD among AYAs. Abbreviations: EAPC = estimated annual percentage change. ADHD = attention deficit hyperactivity disorder. AYAs = adolescents and young adults.

At the national level, Fig 5 summarizes the global prevalence and EAPC for ADHD in AYAs in 2021. Overall, the prevalence increased the most in the UK, with an annual increase of 0.54% (95% CI: 0.45 to 0.63), while Thailand saw the largest decline (−0.34%, 95% CI: −0.41 to −0.27). After considering the weighted APCs, China exhibited the highest AAPCs (18.20%, 95% CI: 16.12 to 20.28), while Finland had the lowest AAPCs (−13.40%, 95% CI: −14.46 to −12.33).

**Fig 6:**
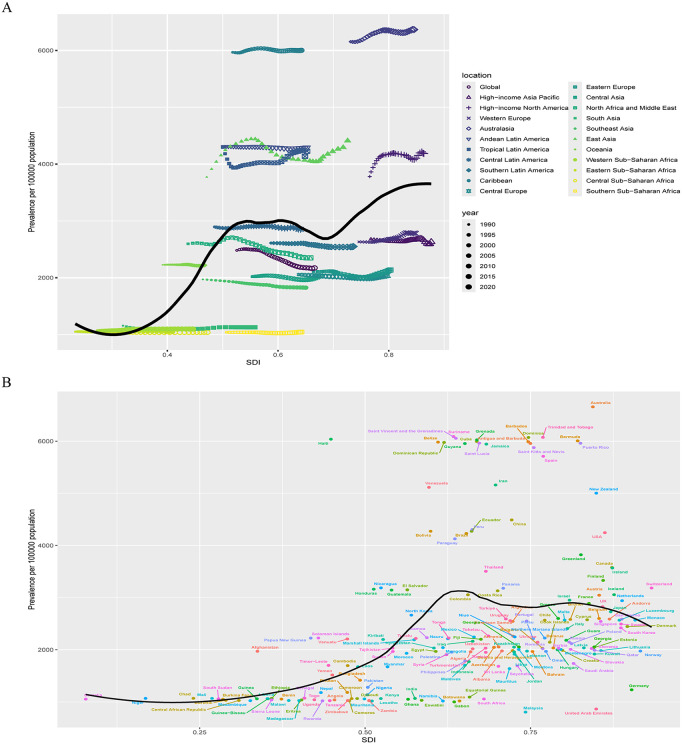
The association between the prevalence of ADHD among AYAs and SDI at the regional level from 1990 to 2021 (A) and national level at 2021 (B). Abbreviations: ADHD = attention deficit hyperactivity disorder. AYAs = adolescents and young adults. SDI = socio-demographic index.

Regarding the relationship between prevalence and SDI regions, a nonlinear pattern emerged. Prevalence increased significantly from low SDI to middle SDI regions, but the rise from middle to high SDI regions was less pronounced, showing irregular fluctuations (Fig 6A). Between 1990 and 2021, regions like East Asia, High-Income North America, Tropical Latin America, Andean Latin America, Australasia, and the Caribbean had higher-than-expected prevalence, with East Asia exhibits the most significant fluctuations, consistently remaining above expected levels. In addition, Australasia and the Caribbean region report the highest prevalence rates, all of which also exceed anticipated figures. Conversely, regions like Western Europe, High-Income Asia Pacific, South Latin America, Eastern Europe, Central Europe, Central Asia, Southeast Asia, and Southern Sub-Saharan Africa exhibited lower-than-expected prevalence.

At the national level, a similar nonlinear relationship was observed between prevalence and SDI regions (Fig 6B). ADHD burden was high not only in developed countries but also in underdeveloped nations. However, high SDI countries showed more instances of prevalence exceeding expectations compared to low SDI countries, with countries like Australia and Haiti showing the greatest deviations above expected rates, while countries like Malaysia and Germany had the largest deviations below expected rates.

### 3.3 Disability-adjusted Life-years

From 1990 to 2021, the number of DALYs due to ADHD in AYAs increased from 469,027–501,443 globally. The age-standardised DALYs rate decreased slightly from 30.31 per 100,000 population to 26.56 per 100,000 population. The overall trend showed an annual decline of 0.58% (EAPC: −0.58%, 95% CI: −0.63 to −0.52). Locally, the AAPCs undergoing the third joinpoint was −0.44% (95% CI: −0.47 to −0.42), indicating a statistically significant downward trend. The DALYs rate showed an upward trend from 1990 to 1993, followed by varying degrees of decline from 1993 to 2000, 2000–2015, and 2015–2021.

By gender, both male and female AYAs showed a decline in DALYs rates, with males exhibiting higher DALYs rates than females. From 1990 to 2021, males experienced an average annual decline of 0.60% (95% CI: −0.64 to −0.55) in age-standardised DALYs rates, while females showed a slightly slower decline of 0.57% (95% CI: −0.64 to −0.50). Locally, the trends were consistent with the overall AYAs population: males showed a decline with an AAPCs of −0.45% (95% CI: −0.48 to −0.42), while females showed an increase from 1990 to 2000 and declines in subsequent periods, with AAPCs of −0.45% (95% CI: −0.49 to −0.42).

**Fig 7:**
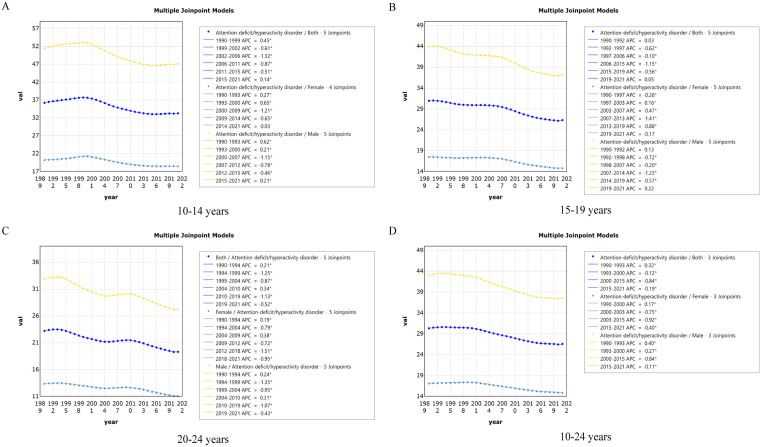
The joinpoint regression analysis on the rate of DALYs of ADHD among 10-14 years (A), 15-19 years (B), 20-24 years (C) and 10-24 years (D) AYAs. Abbreviations:* Indicates that the APCs is significantly different from zero at the alpha = 0.05 level. ADHD = attention deficit hyperactivity disorder. AYAs = adolescents and young adults. DALYs = disability-adjusted life-years.

Regarding age groups, all groups showed a declining trend in DALYs, with the most significant decrease observed in the 20−24 age group, which declined by 0.61% (EAPC = −0.61%, 95% CI: −0.67 to −0.55) annually. The 15−19 age group followed with a 0.59% decline (EAPC = −0.59%, 95% CI: −0.65 to −0.53), while the 10−14 age group showed the least decline (EAPC = −0.48%, 95% CI: −0.57 to −0.40). Local trend analysis confirmed these overall findings, with the AAPCs for the 20−24 age group being the steepest decline (−0.61%, 95% CI: −0.65 to −0.57), and the slowest decrease observed in the 10−14 age group (−0.28%, 95% CI: −0.32 to −0.24) (Fig 7).

DALYs rates varied across SDI regions, in general, a higher SDI is positively associated with an elevated disease DALYs. The overall trend revealed that middle SDI regions had the largest decrease in age-standardised DALYs (EAPC = −0.44%, 95% CI: −0.51 to −0.38), while high SDI regions had the largest increase (EAPC = 0.21%, 95% CI: 0.17 to 0.25). Local analysis showed the lowest AAPCs in middle SDI regions (−0.10%, 95% CI: −0.10 to −0.09), while high SDI regions had the highest AAPCs (0.11%, 95% CI: 0.11 to 0.12).

Regionally, Western Europe exhibited the highest increase in age-standardised DALYs, with an annual increase of 0.23% (EAPC = 0.23%, 95% CI: 0.21 to 0.25), while North Africa and Middle East showed the largest decrease (EAPC = −0.40%, 95% CI: −0.49 to −0.32). Locally, East Asia had the highest AAPCs (0.22%, 95% CI: 0.19 to 0.24), while North Africa and Middle East had the lowest AAPCs (−0.10%, 95% CI: −0.10 to −0.10) (Table 3).

**Table 3:** The case number and rate of DALYs of ADHD among AYAs in 1990 and 2021 by SDI quintiles and by GBD regions,with EAPC and AAPCs from 1990 to 2021.

	1990		2021		EAPC (95% CIs)	AAPCs (95% CIs)
	Number (95% UIs)	Rate (95% UIs)	Number (95% UIs)	Rate (95% UIs)		
**Global**	469026.9 (247085.21 to 776225.78)	30.31 (15.97 to 50.17)	501443.81 (262431.34 to 823453.05)	26.56 (13.9 to 43.62)	−0.58 (−0.63 to −0.52)	−0.44 (−0.47 to −0.42)
**Sex**						
Male	338465.26 (177626.81 to 558946.14)	43.06 (22.6 to 71.1)	363937.96 (188861.83 to 595965.50)	37.62 (19.52 to 61.6)	−0.60 (−0.64 to −0.55)	−0.45 (−0.48 to −0.42)
Female	130561.64 (68557.29 to 216892.26)	17.15 (9.01 to 28.5)	137505.85 (72895.48 to 230419.96)	14.94 (7.92 to 25.04)	−0.57 (−0.64 to −0.5)	−0.45 (−0.49 to −0.42)
**Age group**						
10-14 years	194008.69 (99206.51 to 337758.52)	36.22 (18.52 to 63.05)	221885.56 (112888.86 to 383739.82)	33.28 (16.93 to 57.56)	−0.48 (−0.57 to −0.40)	−0.28 (−0.32 to −0.24)
15-19 years	160828.32 (84092.22 to 265666.75)	30.96 (16.19 to 51.15)	164273.35 (85802.74 to 270646.01)	26.33 (13.75 to 43.37)	−0.59 (−0.65 to −0.53)	−0.53 (−0.58 to −0.48)
20-24 years	114189.87 (61213.37 to 187683.17)	23.21 (12.44 to 38.14)	115284.90 (62479.51 to 189562.86)	19.31 (10.46 to 31.74)	−0.61 (−0.67 to −0.55)	−0.61 (−0.65 to −0.57)
**GBD regions**						
Central Europe	7349.18 (3768.61 to 12090.32)	25.17 (12.91 to 41.41)	4543.78 (2312.94 to 7475.27)	25.05 (12.75 to 41.22)	−0.04 (−0.07 to 0)	−0.00 (−0.00 to-0.00)
East Asia	172096.82 (90568.38 to 285831.81)	46.24 (24.33 to 76.8)	131650.15 (70998.45 to 211940.53)	54.17 (29.22 to 87.21)	0.01 (−0.14 to 0.16)	0.22 (0.19 to 0.24)
South Asia	47011.82 (24552.58 to 78997.41)	14.06 (7.34 to 23.62)	72343.52 (37192.44 to 120607.5)	13.76 (7.07 to 22.93)	0.04 (−0.01 to 0.1)	−0.01 (−0.01 to −0.01)
Western Europe	26393.9 (14334.24 to 43097.63)	32.11 (17.44 to 52.43)	24428.69 (12711.18 to 39884.36)	33.89 (17.64 to 55.34)	0.23 (0.21 to 0.25)	0.06 (0.06 to 0.06)
Tropical Latin America	24993.97 (12921.68 to 42170.63)	52.22 (27 to 88.11)	25996.17 (13403.83 to 43326.8)	51.4 (26.5 to 85.67)	0.15 (0.07 to 0.23)	−0.03 (−0.05 to −0.02)
High-income North America	28225.17 (14759.71 to 47337.39)	46.14 (24.13 to 77.38)	36343.86 (18372.8 to 61744.64)	50.99 (25.78 to 86.62)	0.14 (0.07 to 0.22)	0.17 (0.15 to 0.18)
Oceania	568.21 (299.47 to 959.99)	27.16 (14.32 to 45.89)	1092.42 (571.77 to 1853.43)	27.09 (14.18 to 45.96)	−0.01 (−0.02 to 0)	−0.00 (−0.00 to 0.00)
Central Latin America	19181.9 (9991.8 to 32028.73)	35.35 (18.42 to 59.03)	22655.89 (11839.34 to 37595.13)	34.84 (18.21 to 57.81)	−0.01 (−0.03 to 0.01)	−0.02 (−0.02 to −0.01)
Caribbean	7758 (4211.12 to 12614.4)	72.65 (39.43 to 118.13)	8279.53 (4471.17 to 13911.95)	73.09 (39.47 to 122.82)	0.01 (0 to 0.03)	0.02 (0.01 to 0.02)
High-income Asia Pacific	13828.73 (6973.08 to 23053.2)	32.83 (16.55 to 54.72)	8407.47 (4236.86 to 13826.21)	32.21 (16.23 to 52.96)	−0.02 (−0.06 to 0.01)	−0.02 (−0.02 to −0.01)
Southeast Asia	35747.3 (18182.59 to 59930.03)	24.09 (12.26 to 40.39)	38196.96 (19361.1 to 64740.97)	22.34 (11.32 to 37.86)	−0.25 (−0.26 to −0.24)	−0.06 (−0.06 to −0.06)
Central Asia	4891.55 (2487.87 to 7973.57)	24.67 (12.55 to 40.21)	5508.65 (2806.99 to 9067.5)	24.9 (12.69 to 40.98)	−0.08 (−0.12 to −0.03)	0.01 (0.01 to 0.01)
Southern Latin America	4216.54 (2196.67 to 7093.34)	31.85 (16.6 to 53.59)	4777.59 (2507.87 to 7844.91)	31.15 (16.35 to 51.15)	−0.11 (−0.12 to −0.1)	−0.02 (−0.03 to −0.02)
North Africa and Middle East	34497.7 (17799.11 to 56980.7)	31.68 (16.34 to 52.32)	46607.98 (24548.1 to 76756.61)	28.72 (15.13 to 47.29)	−0.4 (−0.49 to −0.32)	−0.10 (−0.11 to −0.10)
Eastern Europe	12177.83 (6203.59 to 20632.3)	25.78 (13.14 to 43.69)	8629.02 (4445.11 to 14655.96)	26.15 (13.47 to 44.42)	−0.05 (−0.13 to 0.02)	0.02 (0.01 to 0.02)
Australasia	3606.77 (1929.61 to 5915.6)	74.97 (40.11 to 122.96)	4455.22 (2411.38 to 6946.37)	77.66 (42.03 to 121.08)	0.11 (0.08 to 0.13)	0.09 (0.08 to 0.10)
Andean Latin America	6481.26 (3368.15 to 10806.38)	52.65 (27.36 to 87.78)	9036.78 (4691.29 to 15136.98)	52.34 (27.17 to 87.68)	−0.02 (−0.03 to −0.02)	−0.01 (−0.01 to −0.01)
Western Sub-Saharan Africa	7771.66 (4057.27 to 13387.46)	12.99 (6.78 to 22.37)	21559.22 (11237.91 to 36124.29)	13.36 (6.96 to 22.39)	0.12 (0.1 to 0.14)	0.01 (0.01 to 0.01)
Eastern Sub-Saharan Africa	7896.59 (4094.42 to 13191.67)	12.73 (6.6 to 21.27)	18472.74 (9751.31 to 30959.17)	12.7 (6.7 to 21.29)	0.01 (0 to 0.02)	−0.00 (−0.00 to −0.00)
Central Sub-Saharan Africa	2168.43 (1121.09 to 3618.67)	12.53 (6.48 to 20.91)	5679.67 (2920.24 to 9490.41)	12.64 (6.5 to 21.12)	0.02 (0.01 to 0.03)	0.00 (0.00 to 0.01)
Southern Sub-Saharan Africa	2163.53 (1139.39 to 3675.02)	12.66 (6.67 to 21.51)	2778.51 (1438.89 to 4798.43)	12.74 (6.6 to 22)	−0.01 (−0.03 to 0.01)	0.00 (0.00 to 0.00)
**SDI quintiles**						
High-middle SDI	108640.66 (57121.38 to 179336.51)	38.28 (20.13 to 63.19)	90187.99 (48659.66 to 146818.76)	39.93 (21.54 to 65)	−0.18 (−0.26 to −0.09)	0.03 (0.02 to 0.04)
Middle SDI	195229.57 (102406.59 to 322075.12)	35.57 (18.66 to 58.68)	182270.65 (95485.44 to 297797.08)	32.98 (17.27 to 53.88)	−0.44 (−0.51 to −0.38)	−0.10 (−0.10 to −0.09)
Low-middle SDI	70338.52 (36762.26 to 117158.75)	19.45 (10.16 to 32.39)	100857.68 (51789.94 to 167445.08)	18.25 (9.37 to 30.29)	−0.15 (−0.17 to −0.13)	−0.04 (−0.04 to −0.04)
High SDI	71719.36 (37882.87 to 117489.29)	36.62 (19.34 to 59.99)	74490.44 (38537.73 to 122345.95)	40.14 (20.77 to 65.93)	0.21 (0.17 to 0.25)	0.11 (0.11 to 0.12)
Low SDI	22583.29 (11722.58 to 37894.94)	14.51 (7.53 to 24.34)	53106.25 (27704.90 to 88217.46)	14.38 (7.50 to 23.88)	0.03 (0.01 to 0.05)	−0.00 (−0.01 to −0.00)

Abbreviations: DALYs, disability-adjusted life-years. ADHD, attention deficit hyperactivity disorder. AYAs, adolescents and young adults. SDI, socio-demographic index. GBD, Global Burden of Diseases, Injuries, and Risk Factors Study. EAPC, estimated annual percentage change. UIs, uncertainty intervals. CIs, confidence intervals. AAPCs, average annual percentage changes.

**Fig 8:**
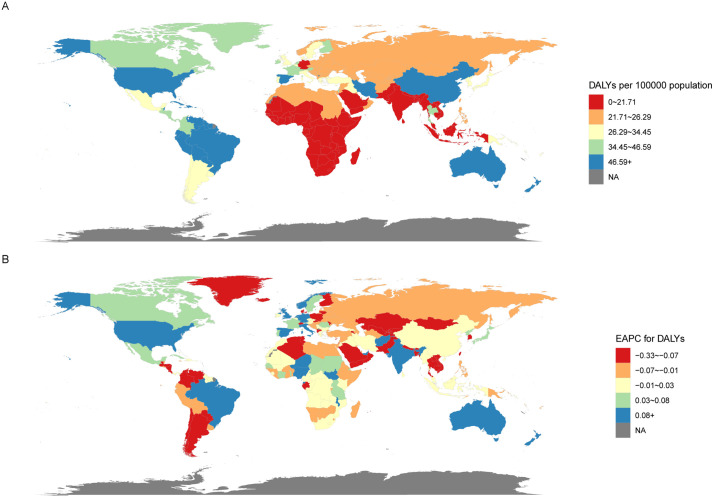
Global map of 2021 DALYs (A) and the trend (EAPC) in DALYs (B) of ADHD among AYAs. Abbreviations: EAPC = estimated annual percentage change. ADHD = attention deficit hyperactivity disorder. AYAs = adolescents and young adults. DALYs = disability-adjusted life-years.

At the national level, Fig 8 summarizes global DALYs rates and EAPC for ADHD in AYAs in 2021. The UK showed the highest increase, with an age-standardised DALYs rate rising by 0.54% (95% CI: 0.46 to 0.63) annually, while Thailand showed the largest decrease (−0.33%, 95% CI: −0.40 to −0.27). Locally, China exhibited the highest AAPCs (0.23%, 95% CI: 0.20 to 0.26), while Finland had the lowest AAPCs (−0.16%, 95% CI: −0.18 to −0.15).

**Fig 9:**
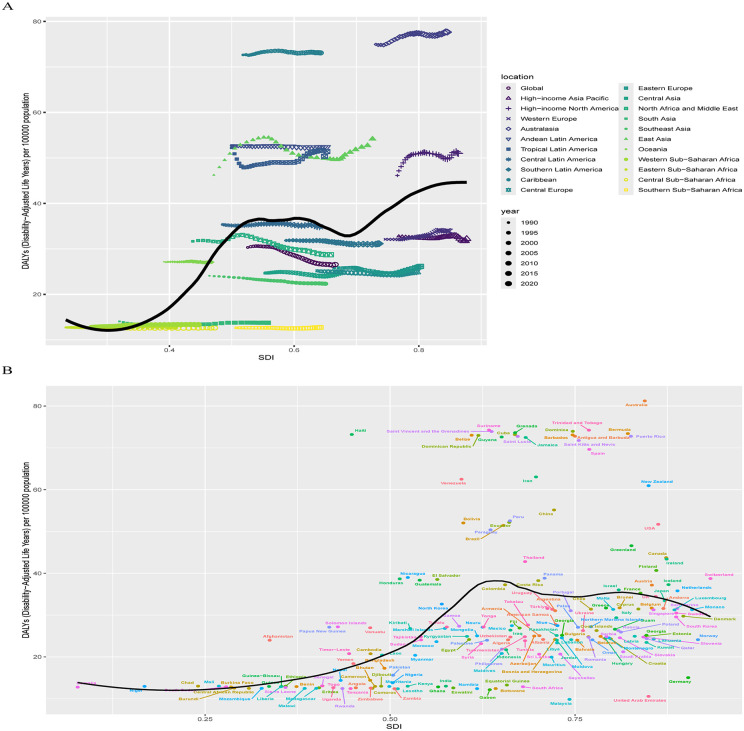
The association between the DALYs of ADHD among AYAs and SDI at the regional level from 1990 to 2021 (A) and national level at 2021 (B). Abbreviations: ADHD = attention deficit hyperactivity disorder. AYAs = adolescents and young adults. SDI = socio-demographic index. DALYs = disability-adjusted life-years.

Regarding the relationship between DALYs and SDI regions, a nonlinear pattern emerged, similar to that seen in prevalence. DALYs rates increased significantly from low SDI to middle SDI regions, but the increase was less pronounced from middle to high SDI regions, showing irregular fluctuations (Fig 9A). Between 1990 and 2021, East Asia, High-Income North America, Tropical Latin America, Andean Latin America, Australasia, and the Caribbean exhibited higher-than-expected DALYs, with East Asia displayed the most substantial fluctuations in DALYs rates, all persistently above expectations, while Australasia and the Caribbean had the highest absolute DALYs rates, both exceeding projected values. Conversely, Western Europe, High-Income Asia Pacific, South Latin America, Eastern Europe, Central Europe, Central Asia, Southeast Asia, and Southern Sub-Saharan Africa showed lower-than-expected DALYs.

At the national level, a similar nonlinear relationship between DALYs and SDI regions was observed (Fig 9B). High ADHD burden was present in both developed and underdeveloped countries, but high SDI countries showed more instances of DALYs exceeding expectations. Countries like Australia and Haiti had the greatest deviations above expected DALYs rates, notably, countries such as Malaysia and Germany exhibited the largest negative deviations from expected DALYs rates.

## 4 Discussion

Based on GBD data, this study presents the latest findings on the incidence, prevalence, and DALYs rates of ADHD in AYAs. To our knowledge, this is the first systematic assessment of the global, regional, and national burden and trends of ADHD among AYAs across 204 countries and regions from 1990 to 2021.

The results show a general decline in the incidence, prevalence, and DALYs rates of ADHD in AYAs globally from 1990 to 2021. Although the overall trend is downward, when broken down into sub-periods, we observed a triphasic pattern of incidence: an initial rise, followed by a decline, and subsequently a resurgence, with a noticeable increase in ADHD incidence starting in 2013. This increase may be linked to the changes in the diagnostic criteria for ADHD in the DSM-5 [15], which was updated in 2013. For example, the age cutoff for symptom onset was raised from 7 to 12 years [16]. This change represented a significant broadening of the diagnostic criteria, allowing individuals with later-onset symptoms to be diagnosed for the first time, which in turn likely contributed to the observed increase in incidence. While the overall 30-year trend shows a decline, this masks a more complex recent pattern. The general decline in DALYs since the 1990s may be partially attributed to advances in therapeutic approaches and management strategies, which can reduce the severity and duration of disability for those who are diagnosed [17]. However, the resurgence in incidence and the slowing pace of decline in prevalence are likely driven by a different, opposing set of factors. These factors include heightened public awareness, changes in diagnostic standards, and improved diagnostic accuracy [18]. These factors do not create other disease, but they reveal a larger pool of previously undiagnosed cases. The rate at which ADHD cases are being diagnosed may be outpacing the rate at which they are being resolved through treatment. This suggests that the global burden of ADHD in the past has most likely been underestimated, and with more precise detection, ADHD’s global burden should increase over time. In summary, ADHD remains a significant concern for AYAs aged 10–24, requiring targeted research and intervention.

Gender analysis showed that, similar to previous studies by Willcutt EG [19] and Joanna Martin [20], males had higher incidence, prevalence, and DALYs rates compared to females. Several studies suggest that gender differences may be influenced by diagnostic biases, referral processes [21], and differing diagnostic criteria, which might better apply to males [22]. Sepcifically, The diagnostic criteria for DSM across all editions have consistently emphasised common externalising symptoms such as hyperactivity and impulsivity in boys. However, symptoms like inattention, which are common among girls, are given insufficient weight in diagnostic criteria despite their prevalence. This creates a significant risk of clinicians misattributing them to anxiety or depressive disorders. Such diagnostic bias leads to the systematic underdiagnosis of women, potentially resulting in a severe underestimation of their true burden. Consequently, observed gender disparities in research may exaggerate actual differences in prevalence rates. Additionally, hormonal fluctuations and comorbidities in females could complicate ADHD diagnosis and treatment [23]. However, further research is needed to clarify these differences.

Regarding age groups, the gender influence on trends was generally similar across both males and females. A key finding was that incidence was only observed in the 10–14 age group. For the 15–19 and 20–24 age groups, only prevalence and DALYs were analyzed, with trends showing younger age groups exhibit slower declines in both prevalence and DALYs. This may reflect the chronic nature of ADHD [24] and the difficulty in diagnosing it in adults,for example, despite the estimated prevalence of adult ADHD of 4.4% in the USA, only a minority of patients are recognized and engaged in treatment [25]. More attention is needed in future studies and treatment for the 15–24 age group.

Regionally, higher ADHD burdens were observed in high-income and upper-middle-income regions, with Australasia showing the highest incidence, prevalence, and DALYs rates in 2021. However, attention should not only focus on regions with high burdens but also on those with the greatest increases in disease burden. Across all three metrics of incidence, prevalence, and DALYs, Western Europe consistently demonstrated the highest increases in overall trend analyses. Conversely, East Asia exhibited the highest AAPCs when employing weighted averages of segment-specific APCs in all three measures.

At the national level, Australia had the highest DALYs rate, incidence, and prevalence in 2021, while Spain, the UK, and China showed the largest increases in rates. It is important to interpret the higher burden in high-income regions, such as Western Europe, with caution. This higher prevalence may be partly attributable to greater public awareness, established healthcare systems, earlier diagnostic services, and improved medical treatment standards. Compared to low-income regions, these factors may lead to higher rates of ADHD case detection in high-income areas. Additionally, the higher ADHD burden in high-income regions like Western Europe may be linked to socioeconomic factors such as urbanization, poverty, and income inequality. For instance, a UK study identified economic hardship as the strongest predictor of ADHD [26]. In East Asia and China, the rapid growth in ADHD rates may be associated with more authoritarian parenting styles. Some studies suggest that more authoritarian parenting styles are not a cause of ADHD, but may be associated with a higher likelihood of parents seeking a diagnosis or reporting symptoms that conflict with those high expectations [27].

Analysis of the relationship between SDI and ADHD rates across regions and countries revealed a complex, nonlinear association. The burden of ADHD exists in both developed and underdeveloped countries. Therefore, leaders in these regions should adopt flexible, context-specific strategies to manage ADHD burden effectively.

This study has limitations. First, the data is sourced from the GBD 2021 database, which may have limitations in data quality, comparability, accuracy, and missing data across different regions and countries. Then, The timeframe of this study spans a period of significant revisions to ADHD diagnostic criteria. Changes to these standards have broadened the pool of diagnosable individuals, potentially introducing artificial fluctuations in observed incidence and prevalence trends. The data utilised in this research may not fully eliminate the interference of this bias in analysing long-term trends. Thirdly, this study focuses on adolescents and young adults aged 10–24 years. The findings reflect the disease burden within this specific age cohort only, failing to capture the full childhood onset trajectory or assess the persistent disease burden of ADHD in adults beyond 24 years of age. Fourthly, the risk of misclassification between ADHD and other neurodevelopmental or psychiatric disorders may introduce bias into the findings. This risk may be heightened in settings with limited healthcare resources. Finally, the study assumes segmented linear trends, limiting its capacity to explain causal mechanisms. It struggles to capture the non-linear effects of factors such as policy changes, healthcare accessibility, educational support, and social media on the burden of ADHD.

## 5 Conclusions

Over the past 30 years, global trends in ADHD incidence, prevalence, and DALYs rates have generally declined. However, recent phase-specific studies reveal varying trends, likely influenced by factors such as socioeconomic development, changes in diagnostic criteria, and evolving treatment methods. Therefore, ongoing research to enhance the accuracy and universality of ADHD diagnosis and treatment is crucial for further alleviating the global health burden of ADHD.

## Abbreviations

ADHD: Attention Deficit Hyperactivity Disorder
AYAs: Adolescents and Young Adults
GBD: Global Burden of Diseases
DALYs: Disability-adjusted Life years
UIs: Uncertainty Intervals
EAPC: Estimated Annual Percentage Change
AAPCs: Average Annual Percentage Changes
SDI: Socio-Demographic Index
IHME: Institute for Health Metrics and Evaluation
CIs: Confidence Intervals
APCs: Annual Percentage Changes
BIC: Bayesian Information Criterion.
